# Supplementary material for: Multi‐dataset identification of innovative feature genes and molecular mechanisms in keratoconus
Source: J Cell Mol Med. 2024 Sep 19;28(18):e70079. doi: 10.1111/jcmm.70079 (PMC11412914; doi:10.1111/jcmm.70079)
Supplement: Supplementary file 2 — Material S2. [file JCMM-28-e70079-s002.zip › 7.GSEA analysis/3.DBNDD1/KEGG_GLYCINE_SERINE_AND_THREONINE_METABOLISM.html]

Details for gene set KEGG\_GLYCINE\_SERINE\_AND\_THREONINE\_METABOLISM[GSEA]

|  || Dataset | DBNDD1.group.cls#h\_versus\_l |
| Phenotype | group.cls#h\_versus\_l |
| Upregulated in class | h |
| GeneSet | KEGG\_GLYCINE\_SERINE\_AND\_THREONINE\_METABOLISM |
| Enrichment Score (ES) | 0.26182953 |
| Normalized Enrichment Score (NES) | 0.88357866 |
| Nominal p-value | 0.6857143 |
| FDR q-value | 1.0 |
| FWER p-Value | 1.0 |
Table: GSEA Results Summary

  

Fig 1: Enrichment plot: KEGG\_GLYCINE\_SERINE\_AND\_THREONINE\_METABOLISM      
 Profile of the Running ES Score & Positions of GeneSet Members on the Rank Ordered List

  

| SYMBOL | TITLE | RANK IN GENE LIST | RANK METRIC SCORE | RUNNING ES | CORE ENRICHMENT || 1 | MAOB | na | 696 | 0.415 | 0.0796 | Yes |
| 2 | GNMT | na | 1520 | 0.334 | 0.1362 | Yes |
| 3 | AGXT2 | na | 3670 | 0.225 | 0.1283 | Yes |
| 4 | AOC2 | na | 4742 | 0.188 | 0.1427 | Yes |
| 5 | GLYCTK | na | 5037 | 0.179 | 0.1773 | Yes |
| 6 | BHMT | na | 5048 | 0.179 | 0.2200 | Yes |
| 7 | GAMT | na | 5079 | 0.178 | 0.2618 | Yes |
| 8 | GLDC | na | 8640 | 0.092 | 0.1813 | No |
| 9 | ALAS2 | na | 9283 | 0.081 | 0.1824 | No |
| 10 | SHMT1 | na | 10382 | 0.066 | 0.1666 | No |
| 11 | DLD | na | 11443 | 0.053 | 0.1487 | No |
| 12 | DMGDH | na | 12130 | 0.046 | 0.1399 | No |
| 13 | SRR | na | 13511 | 0.034 | 0.1084 | No |
| 14 | CBS | na | 16188 | 0.015 | 0.0347 | No |
| 15 | GCAT | na | 16298 | 0.014 | 0.0349 | No |
| 16 | MAOA | na | 19119 | -0.005 | -0.0450 | No |
| 17 | PSPH | na | 20729 | -0.019 | -0.0867 | No |
| 18 | SHMT2 | na | 20784 | -0.020 | -0.0835 | No |
| 19 | SDS | na | 20980 | -0.022 | -0.0838 | No |
| 20 | PSAT1 | na | 22299 | -0.035 | -0.1134 | No |
| 21 | AGXT | na | 23856 | -0.054 | -0.1452 | No |
| 22 | PIPOX | na | 24187 | -0.059 | -0.1406 | No |
| 23 | GATM | na | 24453 | -0.062 | -0.1332 | No |
| 24 | ALAS1 | na | 24880 | -0.069 | -0.1289 | No |
| 25 | PHGDH | na | 26119 | -0.090 | -0.1430 | No |
| 26 | DAO | na | 28346 | -0.139 | -0.1737 | No |
| 27 | CHDH | na | 31161 | -0.227 | -0.2003 | No |
| 28 | SARDH | na | 31538 | -0.242 | -0.1531 | No |
| 29 | CTH | na | 31662 | -0.247 | -0.0973 | No |
| 30 | AMT | na | 33334 | -0.356 | -0.0599 | No |
| 31 | AOC3 | na | 33896 | -0.416 | 0.0240 | No |
Table: GSEA details [plain text format]

  

Fig 2: KEGG\_GLYCINE\_SERINE\_AND\_THREONINE\_METABOLISM      
 Blue-Pink O' Gram in the Space of the Analyzed GeneSet

  

Fig 3: KEGG\_GLYCINE\_SERINE\_AND\_THREONINE\_METABOLISM: Random ES distribution      
 Gene set null distribution of ES for **KEGG\_GLYCINE\_SERINE\_AND\_THREONINE\_METABOLISM**
